# The Role of Histology in Predicting Spread Through Air Spaces (STAS) in Non-Small Cell Lung Cancer

**DOI:** 10.3390/jpm16080401

**Published:** 2026-07-27

**Authors:** Marco Chiappetta, Alessandra Cancellieri, Carolina Sassorossi, Filippo Lococo, Teresa Ferrazzo, Chiara Scognamiglio, Alessia Senatore, Adriana Nocera, Qianqian Zhang, Andrea Guarneri, Silvia Taralli, Maria Lucia Calcagni, Flaminia Vocaturo, Luca Boldrini, Huong Elena Tran, Isabella Sperduti, Stefano Margaritora

**Affiliations:** 1 Thoracic Surgery, Magna Graecia University, 88100 Catanzaro, Italy; marcokiaps@hotmail.it (M.C.); teresaferrazzo31@gmail.com (T.F.); 2 Pathology Unit, Fondazione Policlinico Universitario “A. Gemelli” IRCCS, 00168 Rome, Italy; alessandra.cancellieri@policlinicogemelli.it (A.C.); qianqian.zhang@policlinicogemelli.it (Q.Z.); 3 Department of Thoracic Surgery, Fondazione Policlinico Universitario “A. Gemelli” IRCCS, Università Cattolica del Sacro Cuore, 00168 Rome, Italy; filippo.lococo@policlinicogemelli.it (F.L.); kiasco1998@gmail.com (C.S.); alessiasenatore2@gmail.com (A.S.); adriana.nocera91@gmail.com (A.N.);; 4 Unit of Nuclear Medicine, Department of Diagnostic Imaging and Radiation Oncology, Fondazione Policlinico A. Gemelli IRCCS, 00168 Rome, Italy; andrea.guarneri@policlinicogemelli.it (A.G.); silvia.taralli@policlinicogemelli.it (S.T.); 5 Section of Nuclear Medicine, Department of Radiological Sciences and Hematology, Università Cattolica del Sacro Cuore, 00168 Rome, Italy; 6 Gemelli Advanced Radiotherapy Center, Fondazione Policlinico Universitario “A. Gemelli” IRCCS, 00168 Rome, Italy; 7 Radiomics GSTeP Core Research Facility, Fondazione Policlinico Universitario “A. Gemelli” IRCCS, 00168 Rome, Italy; 8 Biostatistics, IRCCS Regina Elena National Cancer Institute, 00144 Rome, Italy; isabella.sperduti@ifo.it

**Keywords:** STAS, NSCLC, histology, PET/CT

## Abstract

**Background:** Spread through air spaces (STAS) is a prognostic factor for survival in non-small cell lung cancer (NSCLC), but the chance to identify it before surgery remains challenging. In this study, we consider clinical, pathological and metabolic factors, with the aim to identify possible STAS predictors in NSCLC. **Methods**: Clinical and pathological characteristics of patients who underwent anatomical lung resection from 1 January 2018 to 31 December 2023 were retrospectively reviewed and analyzed. Patients with GGO, AIS, or MIA tumors, metastases, or undergoing neoadjuvant therapy were excluded. The parameters assessed by ^18^F-FDG PET/CT were: SUVmax, SUVmean, SUVpeak, TLG and MTV. The primary endpoint was the association between clinical, metabolic, and pathologic characteristics with the presence of STAS. A univariate and multivariate logistic regression model was developed to identify independent predictors of STAS. **Results:** The final analysis was conducted on 224 patients. Non-lepidic-acinar adenocarcinomas showed a statistically significant higher risk of STAS than the lepidic-acinar histotype: 20.8% vs. 4.3%, *p* = 0.003, OR 5.87, 95%CI 1.83–18.78. Furthermore, tumor grade was significantly associated with STAS: 4.6% in G1–G2 tumors vs. 23.5% in G3 tumors (*p* = 0.001, OR 5.79, 95%CI 2.12–15.78); as well as lymph vascular invasion (*p* = 0.034) and tumor size >2 cm (*p* = 0.017). Multivariate analysis confirmed high tumor grade as an independent risk factor (OR 4.73, 95%CI 1.16–19.23, *p* = 0.030). **Conclusions:** In our study, risk of STAS is not correlated with metabolic parameters, while adenocarcinoma subtypes and tumors grading seem to stratify the risk of STAS occurrence. These results may be consolidated in an external validation analysis to possibly better plan the extent of lung resection.

## 1. Introduction

The definition of spread through air spaces (STAS) was established in the 2015 World Health Organization (WHO) classification [1]. STAS is defined as a lesion in which neoplastic cells from a lung tumor spread into the air spaces of the adjacent lung parenchyma beyond the tumor margin, in the form of micropapillary clusters, solid nests, or single cells [2]; this process does not follow the classic pathways of spread, such as lymph vascular or pleural dissemination, and therefore behaves insidiously.

STAS has recently been validated as a prognostic factor for survival in NSCLC; its presence is particularly common in lung tumors undergoing surgical resection (20–40%) and is associated with a worse prognosis in terms of survival for all histological types [3,4,5]. This is due to a higher risk of locoregional recurrence, which is mainly found in sublobar resections (wedge or segmentectomy), as the surgical resection margin is smaller, and cells can more easily overcome it in the presence of STAS, thereby increasing the risk of recurrence [6,7].

It consequently follows that an accurate preoperative prediction would be helpful in selecting the most appropriate anatomical resection, planning lymph node dissection, choosing wider resection margins and evaluating possible adjuvant treatments.

However, the ability to detect it before surgery remains a critical issue. Currently, diagnosis is based on postoperative histopathological examination rather than preoperative biopsies (percutaneous or bronchoscopic), which are limited by the small amount of material collected, a high risk of false negatives due to the sampling area (STAS is a peripheral phenomenon, whereas biopsies are usually performed in the central area of the lesion), artefacts resulting from the procedure and the absence or limited representation of tissue architecture [8].

Furthermore, radiological imaging techniques only provide indirect and non-specific signs, and they lack reproducibility due to variability in protocols and reconstructions across different centers [8,9,10]; in recent years, however, predictive models based on machine learning and deep learning have emerged that combine CT features and clinical data. These models are showing promise, but they are not yet accurate enough or validated [11,12].

Although the prognostic role of STAS in NSCLC is now well recognized, currently available diagnostic methods do not allow for its reliable identification in the preoperative phase.

Therefore, the aim of this study is to identify possible STAS predictors based on clinical, pathological and metabolic factors in patients affected by non-small cell lung cancer.

## 2. Methods

The study received approval from the Institutional Ethics Committee (No. 0003291/23) and was conducted in compliance with the principles outlined in the Declaration of Helsinki.

Clinical and pathological data from patients diagnosed with NSCLC who underwent anatomical lung resection at our institution between 1 January 2018 and 31 December 2023 were retrospectively reviewed and analyzed.

### 2.1. Inclusion and Exclusion Criteria

The study population was selected according to the following criteria.

Inclusion Criteria

- Histologically confirmed NSCLC
- Presence of solid or subsolid pulmonary lesions
- Complete surgical lung resection
- Age greater than 18 years
- Availability of a preoperative ^18F-FDG PET/CT examination
- Preoperative evaluation by a multidisciplinary tumor board

Exclusion Criteria

- Pure ground-glass opacity (GGO) nodules
- Atypical adenomatous hyperplasia (AAH), adenocarcinoma in situ (AIS), or minimally invasive adenocarcinoma (MIA)
- Evidence of distant metastatic disease
- Receipt of neoadjuvant therapy
- Missing or unavailable follow-up data

### 2.2. Preoperative Assessment

All patients underwent a comprehensive preoperative evaluation, including contrast-enhanced computed tomography (CT) of the brain, chest, and abdomen (with brain magnetic resonance imaging when clinically indicated), ^18^F-FDG PET/CT imaging, pulmonary function testing, laboratory investigations, and cardiovascular assessment.

### 2.3. Surgical Procedure

Operations were performed by board-certified thoracic surgeons using either minimally invasive approaches or open thoracotomy, depending on tumor stage and surgeon expertise.

### 2.4. Pathological Evaluation

Surgical and pathological records were reviewed to collect information regarding tumor histology, spread through air spaces (STAS), pleural invasion, lymphovascular invasion, and tumor necrosis. STAS was defined as the presence of micropapillary clusters, solid nests, or isolated tumor cells extending into the air spaces of the adjacent lung parenchyma beyond the margin of the primary tumor, in accordance with previously published criteria [13].

### 2.5. PET/CT Analysis

Metabolic parameters, including maximum standardized uptake value (SUVmax), mean standardized uptake value (SUVmean), peak standardized uptake value (SUVpeak), total lesion glycolysis (TLG), and metabolic tumor volume (MTV), were measured from primary tumor lesions by experienced nuclear medicine physicians using Syngo.via (Siemens Healthineers) software, syngo.via VB80.

Tumor segmentation was performed semi-automatically using a threshold corresponding to 40% of SUVmax, as previously reported in the literature [14]. Manual adjustment of PET contours was carried out whenever deemed necessary.

### 2.6. Histopathological Review

Two experienced pathologists independently reviewed all specimens and documented the presence of STAS, pleural invasion, lymphovascular invasion, and necrosis.

### 2.7. Adjuvant Treatment and Follow-Up

Patients with pathologically confirmed lymph node involvement received adjuvant therapy according to current clinical guidelines [15,16].

Follow-up assessments included clinical examination, laboratory testing, and thoracic CT or brain–thorax–abdomen imaging every 6 to 12 months for a period of five years, with surveillance schedules tailored according to tumor stage and histological subtype.

### 2.8. Statistical Analysis

The primary endpoint of the study was to evaluate the association between clinical, metabolic, and pathological variables and the presence of STAS.

Odds ratios (ORs) with corresponding 95% confidence intervals (95% CIs) were calculated for each variable of interest. Statistical significance was defined as a two-sided *p*-value < 0.05.

To identify independent predictors of STAS, a multivariable logistic regression model was constructed using a stepwise forward selection approach. Variables were entered and removed from the model according to predefined thresholds of *p* = 0.10 and *p* = 0.15, respectively.

## 3. Results

The final analysis was conducted on 224 patients who met the inclusion/exclusion criteria. Histology consisted in adenocarcinoma in 164 (72.3%) patients, while stage Ia was present in 160 (71.4%) cases (Table 1). STAS was detected in 24 (10.7%) cases.

### 3.1. STAS Risk in the Entire Cohort

Patient characteristics, stage, nodal status and metabolic parameters did not correlate with the presence of STAS (Table 2), while pathological characteristics did.

Univariable analysis identified increased risk for STAS in G3 tumors (*p* = 0.001) and non-acinar/lepidic histology (*p* = 0.003), while lymphvascular invasion was nearly statistically significant (*p* = 0.053).

In detail, STAS was present in the 23.5% of G3 tumors vs 4.6% in G1–G2 (*p* = 0.001, OR 5.79, 95%CI 2.12–15.78) and in the 20.8% of non-acinar/lepidic histology vs. 4.3% of acinar/lepidic histology (*p* = 0.003, OR 5.87, 95%CI 1.83–18.78).

Multivariable analysis confirmed tumor grading as an independent prognostic factor: OR 5.44, 95%CI 1.75–16.84, *p* = 0.003.

### 3.2. Risk of STAS in Stage IA

Considering stage Ia tumors only, STAS occurred in 20 cases (12.5%). Non-acinar/lepidic histology (*p* < 0.001), high tumor grading (*p* = 0.001), lymphovascular invasion (*p* = 0.034) and pT > 2 cm (*p* = 0.017) results significantly correlated with the presence of STAS from the univariable analysis (Table 3).

STAS was present in 4.9% of G1–G2 vs. 20.2% G3 tumors, in 8.9% of tumors ≤2 cm vs 24.3% of tumors > 2 cm, in 29.4% of cases with lymphovascular invasion vs. 11.4% without it, and in 29.8% of non-acinar/lepidic histology adenocarcinoma vs. 2.7% of acinar/lepidic histology, occurring only in 2 of the 74 patients with lepidic or acinar histology.

Multivariable analysis adjusted for pT dimension and lymphovascular invasion did not confirm adenocarcinoma subtype and were graded as independent prognostic factors.

## 4. Discussion

STAS was defined as the presence of micropapillary clusters, solid nests, or single tumor cells that extend into the air spaces of the surrounding lung parenchyma beyond the edge of the main tumor [13]. It represents a newly recognized form of tumor infiltration since the 2015 WHO Classification of lung tumors besides traditional forms, such as lympho-vascular, stromal, and pleural invasion, and it is associated with a high risk of locoregional recurrence in adenocarcinoma as well as in other histotypes, including squamous cell carcinoma, neuroendocrine neoplasms, and pleomorphic carcinoma [17,18,19]. According to the literature, its presence therefore precludes a diagnosis of the main variants of low-grade adenocarcinoma, such as minimally invasive adenocarcinoma (MIA) and adenocarcinoma of low malignant potential [3,20].

In this study, we evaluated the risk of STAS according to clinical and pathological factors in patients who had undergone surgical resection for NSCLC.

One of our main findings is the independent association between the risk of STAS and non-acinar/lepidic adenocarcinoma in the entire cohort and also in stage I, reflecting the importance of obtaining this information prior to or during surgery. Indeed, in the approach to NSCLC treatment, the diagnosis could be preoperative, through percutaneous or endoscopic biopsy, or intra-operative, with frozen section.

Although these two possible approaches may give histological information, both lack accuracy in determining the presence of STAS.

The benefit of a preoperative biopsy is limited because it usually misses tumor architecture and does not permit assessment for the presence of clusters, nests or single cancer cells inside the alveolar spaces; on the other hand, frozen sections can include alveolar spaces and therefore allow the assessment of STAS, but artifacts and other factors can be present to limit the visualization of STAS [21,22].

Walts et al. [23], in consideration of the role of frozen sections in the assessment of STAS, reported an excellent positive predicted value of 100% but a very insufficient negative low prognostic value of 8%, while STAS was often detected in the tissue adjacent to the tumor in permanent sections. Morimoto et al. [24] stated that another limit in determining STAS in frozen sections was due to inadequate inflation of the lung, which did not permit assessment of the presence of clusters of tumor cells in the alveolar spaces.

On the other hand, both pre-operative biopsy and frozen sections allow researchers to obtain information about the two main determinants of STAS, as shown in the present study: histology, adenocarcinoma subtype, and tumor grading.

Currently, STAS is a validated prognostic factor in NSCLC reported in nearly all histotypes [8], but it could also be a determinant in choosing the appropriate surgical resection. Indeed, different studies reported a worse prognosis in STAS-positive patients who underwent segmentectomy compared to lobectomy, even in stage I [4,25,26], although some observations should be taken in account. In the paper of Pan et al. [26], this prognostic difference among parenchymal resections was present in stages higher than IA1, suggesting that in centimetric tumors, segmentectomy may be equivalent to lobectomy even in the presence of STAS. This surgical indication may also be confirmed in pure ground-glass or part-solid tumors, which present a low risk of STAS [25,27,28,29], perhaps due to the high incidence of lepidic patterns rarely associated with STAS.

Indeed, lepidic pattern is associated with STAS in a very low percentage of cases, (about 0–2%) [29,30,31], with the highest rate of 50% reported by Huang et al., but nevertheless inferior to the percentages reported in other adenocarcinoma subtypes [25]. In our cohort, lepidic growth was associated with a lower incidence of STAS, consistent with previous reports, suggesting a more favorable biological behavior and supporting the consideration of parenchymal-sparing resections, such as segmentectomy, in appropriately selected patients.

This result needs to be clarified; although adenocarcinoma subtypes showed a significant association with STAS in the univariable analysis, this association was not retained in the multivariable model. This finding should not be interpreted as evidence that adenocarcinoma subtype is unrelated to STAS. Rather, it suggests that its apparent effect may be influenced by other clinicopathological variables included in the model. The discrepancy between univariable and multivariable results may reflect confounding collinearity among predictors, mediation effects, or limited statistical power. Therefore, while adenocarcinoma subtype may contribute to the identification of patients at increased risk of STAS, our findings do not support its role as an independent predictor in the adjusted analysis.

Furthermore, even though this is an important association, confirming data already present in literature, we are aware that this result would not help in planning extension of the resection, as both the adenocarcinoma subtype and tumor grading are both defined after surgery.

The correlation between GGO/part-solid nodules and lepidic growth is validated, and available literature confirms that STAS is very uncommon in this setting. Similarly, solid nodules presenting lepidic patterns may also be taken advantage of from sublobar resections instead of lobectomy, considering their low aggressiveness and the limited risk of STAS. In this regard, it should be possible to evaluate the risk of STAS according to the radiological appearance of the nodule but also from pre-op biopsy or frozen section for as much as pre-op biopsies may give enough information about the possible histotype, frozen sections, especially if evaluated by expert lung pathologists, may give accurate information about histotype, grading, or presence of invasion. The sum of the histological and radiological information could lead to choosing more appropriate lung resections according to the effective risk of STAS in each case.

A limitation, however, could be linked to the different experience and skills of the pathologists involved. Indeed, although there is usually much agreement among pathologists about the definition of STAS, there are still many areas of controversy it i the recognition of artifacts, leading to the possibility of different interpretations and STAS recognition [32]. These critical issues, reported during specimen analysis, can be sharpened during frozen section evaluation, suggesting the need of other indirect factors to confirm the presence of STAS, and therefore the knowledge of the pattern of growth and the tumor grading may be extremely useful in this scenario.

Interestingly, histological information seems to be essential especially in case of tumors between 1 and 2 cm, because this is the critical tumor size indicated for segmentectomy, and STAS prediction in this setting is limited.

As previously reported, in tumors <1 cm, segmentectomy seems to be equivalent to lobectomy in survival terms, while in tumors >1 cm with STAS segmentectomy seems to ensure a worse prognosis compared to lobectomy. Our study, in agreement with available literature, confirmed that the risk of STAS is increased in cases of tumors >2 cm [25,27,32] and, therefore, in this setting, lobectomy should be the intervention of choice to ensure the best survival possibility. This would also be in agreement with the findings of Schuchert et al. [33], who reported the role of lobectomies in taking into account the margins. When tumors do not conform to discrete segmental boundaries, or when a reasonable margin cannot be obtained, lobectomy should be considered.

On the other hand, in our study, metabolic parameters did not correlate with the presence of STAS. The association between metabolic parameters and STAS is quite controversial in literature, with some studies reporting an increased risk [28,31] according to increased SUV but not according to other parameters such as MTV. Moreover, Nishimori et al. [31] reported a significant association with SUVmax in part-solid tumors only, while no differences were found in solid nodules. Considering the low risk of STAS in part-solid or GGO nodules, the use of metabolic parameters should be limited, while the identification of STAS predictors in solid nodules remains essential.

Similarly to our findings, other studies [25] did not report that SUVmax may predict the presence of STAS, even if it could be strictly correlated to tumor grading and G3 tumors.

Conversely, other studies reported radiomics analysis with encouraging results for STAS prediction [30], which may be integrated and used in clinical settings for resection or neo-adjuvant treatments planning.

This study presents some limitations, based on its retrospective nature and the study period that included years without systematic STAS evaluation. To reduce these limitations, all cases were reviewed by two pathologists, and STAS presence was newly considered. Furthermore, we are aware that the small number of node-positive patients does not allow us to draw definite conclusions.

Future research should focus on the development and validation of reliable preoperative models for STAS prediction, integrating clinical, radiological, metabolic, and histopathological variables. Although tumor size emerged as a potential predictor in the present study, its predictive performance alone is unlikely sufficient for clinical decision making. In this context, radiomics and artificial intelligence-based image analysis may provide additional information beyond conventional imaging assessment, particularly in solid nodules where the risk of STAS is higher and treatment planning remains challenging.

Moreover, prospective multicenter studies with standardized pathological assessment are needed to improve the reproducibility of STAS identification and to reduce interobserver variability, especially in distinguishing true STAS from processing-related artifacts.

## 5. Conclusions

This study showed that STAS risk may potentially be predicted using clinical and pathological information, also in the presence of early-stage lung cancer and in particular, lung adenocarcinoma. Adenocarcinoma subtypes and grading are both collected post-operatively, which may not lead to surgery planning, but tumor dimension is a pre-operative variable that may become useful to predict STAS before surgery. The potential role of adenocarcinoma subtype in STAS risk assessment should be interpreted with caution and warrants further investigation in larger studies. External analysis of a greater cohort may help in the validation of our findings.

## Figures and Tables

**Table 1:** Clinical and pathologic characteristics.

Variable	N (%) or Median
SEX	
male	126 (56.1)
female	98 (43.9)
AGE	71 (40–86) years
SMOKER	
yes	63 (27.9)
no	161 (72.1)
LOBE	
upper	151 (67.4)
lower	73 (32.6)
SIDE	
right	136 (60.7)
left	88 (39.3)
Type of resection	
Lobectomy	161 (71.2)
Segmentectomy	23 (10.5)
Wedge resection	40 (18.3)
Surgical approach	
Uniportal VATS	183 (81.3)
Thoracotomy	41 (18.7)
Margin status	
R0	220 (98.2)
R1	4 (1.8)
Median of dissected nodes (IQR)	
N1	2 (0–6)
N2	4 (1–8)
Total dissected nodes mean ± SD	
N1	3.8 ± 4.2
N2	5.2 ± 5.4
pT	
1a	44 (19.8)
1b	93 (41.2)
1c	44 (19.8)
2a	24 (10.7)
2b	8 (3.6)
3	5 (2.2)
4	6 (2.7)
pT	
1	181 (80.8)
2	32 (14.3)
3–4	11 (4.9)
pT size	
≤2 cm	136 (60.7)
>2 cm	89 (39.3)
HISTOLOGY	
adenocarcinoma	164 (73.2)
squamous cell carcinoma	34 (15.2)
other	26 (11.5)
Adenocarcinoma subtype	
acinar-lepidic	74 (45.1)
other (micropapillary, cribriform, papillary, solid)	90 (54.8)
GRADING	
1–2	135 (60.2)
3	70 (31.2)
Not applicable	19 (8.6)
Pathological N	
0	203 (90.6)
1	7 (3.1)
2	14 (6.3)
SUVmax	4.9 (0.8–34.3)
SUVpeak	2.9 (0.69–23.9)
SUV mean	2.9 (0.56–21.7)
Metabolic total volume	2.1 (0.14–39.4)
Total Lesion Glycolysis	5.7 (0.19–262)
STAS	
Yes	24 (10.7)
No	200 (89.3)
NECROSIS	
Yes	49 (21.9)
No	175 (78.1)
LYMPHOVASCULAR INVASION	
Yes	27 (12.1)
No	197 (87.9)
VISCERAL PLEURAL INVASION	
Yes	49 (21.9)
No	175 (78.1)

**Table 2:** Univariable and multivariable analysis considering risk factors for STAS presence.

Variable			Risk of Stas	
	STAS − *n* (%)	STAS + *n* (%)	Univariable *p* Value (OR; 95%CI)	Multivariable *p* Value (OR; 95%CI)
SEX female male	91 (92.7%) 109 (86.2%)	7 (7.3%) 17(13.8%)	0.131 (2.03; 0.809–5.138)	
AGE			0.846 (1.005; 0.959–1.053)	
SMOKER yes no	58 (91.9%) 142 (87.9%)	5 (8.1%) 19(12.1%)	0.392 (1.570; 0.559–4.407)	
LOBE upper lower	138 (91.2%) 62 (84.5%)	13 (8.8%) 11 (15.5%)	0.142 (1.904; 0.807–4.493)	
SIDE right left	121 (88.7%) 79 (89.5%)	15 (11.3%) 9 (10.5%)	0.851 (1.088; 0.453–2609)	
pT 1 2 3–4	159 (87.5%) 31 (96.9%) 10 (90.9%)	22 (12.5%) 1 (3.1%) 1 (9.1%)	0.348 REF. 0.740 0.438	
pT size ≤2 cm >2 cm			0.257 (1.635; 0.698–3.829)	
HISTOLOGY adenocarcinoma squamous cell carcinoma other	146 (89.0%) 29 (85.3%) 25 (94.4%)	18 (11.0%) 5 (14.7%) 1 (5.6%)	0.538 Ref. 0.392 0.275	
Adenocarcinoma subtype acinar-lepidic other (micropapillary, cribriform, papillary, solid)			0.003 (5.877; 1.839–18.78795)	NOT SIGNIFICANT
GRADING 1–2 3	130 (95.4%) 50 (75.8%)	5 (4.6%) 16 (24.2%)	0.001 (5.792; 2.126–15.780)	0.003 (5.44, 1.75–16.84)
Pathological N 0 1–2	182 (89.4%) 18 (85.7%)	21 (10.6%) 3 (14.3%)	0.222	
SUVmax			0.947 (1.002; 0.946–1.062)	
SUVpeak			0.894 (0.994; 0.916–1.079)	
SUV mean			0.899 (1.006; 0.914–1.107)	
Metabolic total volume			0.545 (1.011; 0.975–1.050)	
Total Lesion Glycolysis			0.919 (1.000; 0.995–1.005)	
NECROSIS NO YES	154 (88.2%) 45 (91.8%)	20 (11.8%) 4 (8.2%)	0.472 (1.510; 0.491–4.647)	
LYMPHOVASCULAR INVASION YES NO	179 (90.6%) 21 (77.8%)	18 (9.4%) 6 (22.2%)	0.053 (2.762; 0.987–7.728)	
VISCERAL PLEURAL INVASION YES NO	156(88.8%) 44 (89.8%)	19 (11.2%) 5 (10.2%)	0.848 (1.107; 0.391–3.13595)	

**Table 3:** Univariable and multivariable analysis considering risk factors for STAS presence in stage Ia patients.

Variable	Risk of Stas	
	Univariable *p* Value (OR; 95%CI)	Multivariable *p* Value (adj-OR; 95%CI) *
SEX male female	0.140 (2.142; 0.778–5.893)	
AGE	0.764 (1.008; 0.957–1.061)	
SMOKER yes no	0.360 (2.142; 0.778–5.893)	
LOBE upper lower	0.334 (1.610; 0.613–4.229)	
SIDE right left	0.713 (1.202; 0.451–3.200)	
pT size ≤2 cm >2 cm	0.017 (3.273; 1.236–8.663)	
HISTOLOGY adenocarcinoma squamous cell carcinoma other	0.691 0.408 0.416	
Adenocarcinoma subtype acinar-lepidic other (micropapillary, cribriform, papillary, solid)	<0.001 (15.750; 3.380–73.394)	Not significant
GRADING 1–2 3	0.001 (6.767; 2.227–20.560)	0.064 (3.553; 0.928–13.598)
SUVmax	0.380 (1.030; 0.964–1.101)	
SUVpeak	0.412 (1.042; 0.944–1.150)	
SUV mean	0.375 (1.050; 0.943–1.169)	
Metabolic total volume	0.403 (0.915; 0.742–1.127)	
Total Lesion Glycolysis	0.720 (0.996; 0.977–1.016)	
NECROSIS YES NO	0.628 (1.341; 0.409–4.392)	
LYMPHOVASCULAR INVASION YES NO	0.034 (3.556; 1.101–11.484)	
VISCERAL PLEURAL INVASION YES NO	0.350 (1.696; 0.561–5.127)	

* OR Adj for pT size and lymphovascular invasion.

## Data Availability

The original contributions presented in this study are included in the article. Further inquiries can be directed to the corresponding author.

## Abbreviations

STAS	Spread through air spaces
NSCLC	Non-Small Cell Lung Cancer
GGO	Ground-Glass Opacity
AIS	Adenocarcinoma In Situ
MIA	Minimally Invasive Adenocarcinoma
^18^F-FDG	Fluorodeoxyglucose F 18
PET/CT	Positron Emission Tomography/Computed Tomography
TLG	Total Lesion Glycolysis
MTV	Metabolic Total Volume
